# The LINC00477/miR-128 axis promotes the progression of polycystic ovary syndrome by regulating ovarian granulosa cell proliferation and apoptosis

**DOI:** 10.1186/s12958-021-00718-z

**Published:** 2021-02-23

**Authors:** Haijie Gao, Jinna Jiang, Yingying Shi, Jiying Chen, Lijian Zhao, Chenhong Wang

**Affiliations:** 1 Department of Obstetrics and Gynecology, Shenzhen Hospital, Southern Medical University, No. 1333, Xinhu Road, Shenzhen, 518000 China; 2 Department of Reproductive Medicine, Women and Children’s Hospital, School of Medicine, Xiamen University, Xiamen, 361003 China; 3 Department of Obstetrics and Gynecology, The First Affiliated Hospital of Xiamen University, Xiamen, 361003 China; 4 Department of Obstetrics and Gynecology, Shenzhen Longhua District Central Hospital, Shenzhen, 518000 China; 5 Department of Obstetrics, People’s Hospital of Guangxi Zhuang Autonomous Region, Nanning, 530021 China

**Keywords:** LINC00477, miR-128, Polycystic ovary syndrome, lncRNA

## Abstract

**Background:**

Long noncoding RNAs (lncRNAs) participate in the pathogenesis of various human diseases. This study aims to investigate the roles of lncRNA LINC00477 in polycystic ovary syndrome (PCOS), especially the impacts of LINC00477 on the proliferation and migration of human granulosa cells and the related mechanisms.

**Methods:**

qRT-PCR analysis was performed to examine the expression pattern of LINC00477 in serum samples of PCOS patients as well as PCOS animal models. The effect of LINC00477 on the viability and apoptosis of ovarian granulosa cells was detected by MTT and flow cytometry assays. The correlation between LINC00477 and miR-128 was verified by bioinformatics analysis and dual-luciferase reporter and RNA pull-down assays. Finally, rescue assays were performed to analyze the effects of the LINC00477-miR-128 axis on the biological behaviors of granulosa cells.

**Results:**

LINC00477 was significantly upregulated in the serum of PCOS patients as well as PCOS mouse models. LINC00477 overexpression inhibited the proliferation and promoted the apoptosis of granulosa cells, whereas knockdown of LINC00477 yielded the opposite effects. Moreover, miR-128 mimics partially abrogated the effect of LINC00477 on granulosa cells.

**Conclusion:**

LINC00477 may function as a ceRNA to inhibit proliferation and apoptosis of granulosa cells by modulating miR-128 expression.

## Introduction

Polycystic ovary syndrome (PCOS) is a commonly diagnosed reproductive disease with a high incidence rate, and PCOS is also one of the leading causes of infertility worldwide [1–3]. The pathogenesis of PCOS remains unclear, leading to difficulties for the early diagnosis and treatment of the disease [4–6]. Therefore, it is of great importance to further explore the underlying mechanism of PCOS and develop novel methods to improve the therapeutic efficacy of the disease.

In recent years, research on the roles of noncoding RNAs in different diseases has become a hot field. Long noncoding RNAs (lncRNAs) are members of the noncoding RNA family, and lncRNAs are often greater than 200 nucleotides in length. Some lncRNAs are kilobases in length [7]. With in-depth investigations of the underlying mechanism of lncRNAs, researchers found that lncRNAs can regulate various biological events, such as cell proliferation, migration, and differentiation, by affecting gene expression at the posttranscriptional level [8, 9]. In the case of carcinogenesis, lncRNAs are involved in the pathogenesis of many types of diseases [10–13], including PCOS [14, 15]. However, the specific underlying mechanisms remain unknown.

Long intergenic non-protein coding RNA 477 (LINC00477) is a recently identified lncRNA [16]. In a previous recent sequencing analysis, LINC00477 was identified as one of the most significantly upregulated LncRNAs in PCOS patients [17]; however, the underlying mechanism remains unclear. In the present study, we further investigated the roles of LINC00477 in PCOS by performing a series of analyses. Our results may provide a potential novel therapeutic target for treating PCOS.

## Methods

### Study design

First, we performed qRT-PCR analysis to examine LINC00477 expression in serum samples of PCOS patients as well as PCOS animal models. Moreover, the correlation between LINC00477 and miR-128 was verified by bioinformatics analysis, dual-luciferase reporter and RNA pull-down assays. Furthermore, the effects of the LINC00477-miR-128 axis on the proliferation and apoptosis of granulosa cells were determined by MTT and flow cytometry assays.

#### Patients and clinical samples

A total of 90 serum samples were obtained from PCOS patients and healthy controls in Shenzhen Hospital, Southern Medical University between Jan. 2019 and Feb. 2020. All patients were diagnosed with PCOS pathologically, and patients with a history of other severe diseases were excluded from this study. Serum samples were stored immediately after surgery in liquid nitrogen until analysis. Informed consent was obtained from each patient. This study was approved by the ethical committee of Shenzhen Hospital, Southern Medical University.

### Establishment of PCOS mice models

A total of 24 female BALC/mice were randomly divided into the PCOS and healthy groups, with 12 mice in each group. PCOS was induced by subcutaneous injection of dehydroepiandrosterone (DHEA) sulfate for 20 days. Then, mice were sacrificed, and ovarium and serum samples were collected. The samples were stored in liquid nitrogen until needed. The animal study was approved by the ethical committee of Shenzhen Hospital, Southern Medical University.

#### In situ hybridization staining

Paraffin-embedded mouse ovarium samples for PCOS and control mice were stained using the in situ hybridization (ISH) method (BOSTER, Wuhan, China) to detect the expression of lncRNA LINC00477 according to the manufacturer’s instruction. Briefly, the samples were first incubated in hybridization solution which contains the probes for lncRNA LINC00477 probes at 42 °C overnight. On day two, the samples were washed and then incubated by biotin labeled antidigoxin antibodies for 1 h in at 37 °C. Finally, the samples were washed again and then incubated by biotin peroxidase at 37 °C for 30 min at 37 °C. Finally, diaminobenzidine tetrahydrochloride (DAB) solution were added for coloration, and the images were captured by an optical microscope.

#### Cell culture

Human granulosa cells were purchased from Procell Life Science & Technology Co., Ltd. (Wuhan, China). Granulosa cells were cultured in RPMI-1640 medium (Invitrogen, Carlsbad, CA, USA). The medium was supplemented with 10% fetal bovine serum (FBS, Fisher, NY, USA), 100 units/ml penicillin and 100 μg/ml streptomycin, and cells were cultured at 37 °C with 5% CO_2_ and 95% humidity.

#### Transfection

The LINC00477 overexpression plasmid was synthesized by Shanghai GenePharma Co., Ltd. (Shanghai, China). Granulosa cells were transfected with LINC00477 siRNA using Lipofectamine 3000 (Invitrogen, Carlsbad, CA, USA) in accordance with the manufacturer’s instructions. The transfection efficiency was determined by RT-qPCR.

#### RT-qPCR

The miRNeasy Mini kit (Qiagen, Valencia, USA) was used to extract total RNA from tissues and cells in accordance with the manufacturer’s instructions. The concentration and quality of the RNAs were determined using a NanoDrop 2000 (Thermo Fisher, Wilmington, USA). First-strand cDNA was synthesized using the TransScript First-Strand cDNA Synthesis Supermix (Transgen, Beijing, China) in accordance with the manufacturer’s instructions. Briefly, a total of 20 μl reaction solution containing 5 μg RNA, 1 μl Random primer, 10 μl 2x TS reaction mix, 1 μl TransScript® RT /RI Enzyme Mix and RNase-free water were prepared, and incubated at 25 °C for 10 min. Then the samples were incubated at 42 °C for 30 min and finally incubated at 85 °C for 5 min to inactivate the enzymes. Next, RT-qPCR assays were performed using SYBR green qPCR Supermix (Applied Biosystems Life Technologies, Foster, USA) in an ABI Prism 7500 sequence detection system (Applied Biosystems Life Technologies, Foster, USA). The 50 μl PCR reaction solution containing 25 μl SYBR® GreenER™ qPCR SuperMix, 1 μl forward primer, 1 μl reverse primer, 5 μl templates as well as 18 μl DEPC-treated water were prepared, and PCR conditions were as follows: 55 °C for 10 min followed by 40 cycles of 95 °C for 30 s, 55-59 °C 30 s and 72 °C for 42 s. Fold changes in the target genes were calculated using the 2^-ΔΔCt^ method (cycle threshold), and miRNA and lncRNA expression levels were normalized by U6 and GAPDH, respectively. The sequences of the primers were as follow: LINC00477, Forward primer 5′- CGGGATCCAGTCTCTTCTTGCAAGGCCTTTCGC-3′; Reverse primer, 5′- GAATTCCGACCTTAGCCTATTTTCATAAGGC-3′. GAPDH, Forward primer 5′-TGTGGGCATCAATGGATTTGG-3′; Reverse primer 5′-ACACCATGTATTCCGGGTCAAT-3′. miR-128, Forward primer, 5′-GCCGGCGCCCGAGCTCTCTGGCTC-3′; Reverse primer, 5′-TCACAGTGAACCGGTCTCTTT-3′. U6, Forward primer 5′-AAAGCAAATCATCGGACGACC-3′; Reverse primer 5′-GTACAACACATTGTTTCCTCGGA-3′.

#### Cell proliferation assay

The 5-diphenyltetrazolium bromide (MTT) assay was adopted to examine granulosa cell proliferation in different groups. Briefly, cells were seeded on 96-well plates (5 × 10^3^/well) and incubated with 100 μl 0.5 mg/ml MTT for 4 h in an incubator, and the precipitate was dissolved in 150 μl dimethylsulfoxide (DMSO). The optical density value of each well at 570 nm was evaluated after shaking for 10 min.

#### Flow cytometry assay

Forty-eight hours after transfection, the cells of different groups were collected and mixed with 5 μl Annexin-V-fluorescein isothiocyanate (FIPCOS) and 2.5 μl propidium iodide (PI). Cellular apoptosis was determined using a FACSAria Sorter (Becton Dickinson, San Jose, USA). The scatter diagram of the apoptotic cells was distributed as follows: Q3 represents early-stage apoptotic cells (FIPCOS+/PI-) and Q2 represents advanced stage apoptotic cells (FIPCOS+/PI+). The apoptotic rate was calculated as number of cells in Q3 + Q2 divided by the total number of cells.

#### Luciferase activity assay

The LINC00477 cDNA fragment, including microRNA binding sites, was cloned into the pmirGLO plasmid (Promega, Madison, USA). Mutant LINC00477 (pmirGLO-DGCR5-MUT) was also generated as the control. Luciferase reporter plasmid and miR-128 mimics or miR-NC mimics were cotransfected into 293 cells using Lipofectamine 2000. Forty-eight hours after transfection, the relative luciferase activity was examined by luminometry using the Dual-Luciferase Reporter Assay System (Promega).

#### Statistical analysis

Data were expressed as the mean ± SD. The comparisons between two groups or multiple groups were examined by Student’s t-test or one-way analysis of variance, respectively. *P* < 0.05 was considered statistically significant. Statistical analysis was performed using SPSS version 13.0 (Chicago, IL, USA).

## Results

### Increased LINC00477 expression in serum samples of PCOS patients

**Fig. 1:**
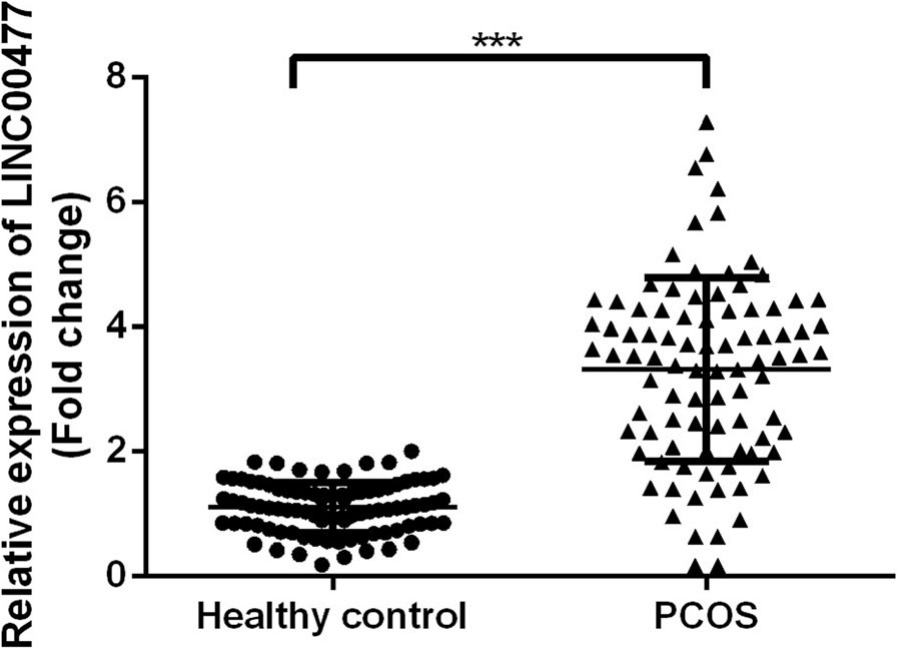
Increased expression of LINC00477 in PCOS. Expression of LINC00477 in serum of PCOS patients and healthy controls. ***p < 0.001 v.s. healthy control

First, 90 serum samples of the patients and healthy controls were collected, and the expression of LINC00477 in serum samples of PCOS patients and healthy controls was compared. We found that LINC00477 expression was markedly increased in the serum of PCOS patients compared with healthy controls. The level of LINC00477 is about 3.3 times higher in the serum of PCOS patients than in the healthy controls (Fig. 1, *p* < 0.001).

### Increased LINC00477 expression in PCOS mouse models

**Fig. 2:**
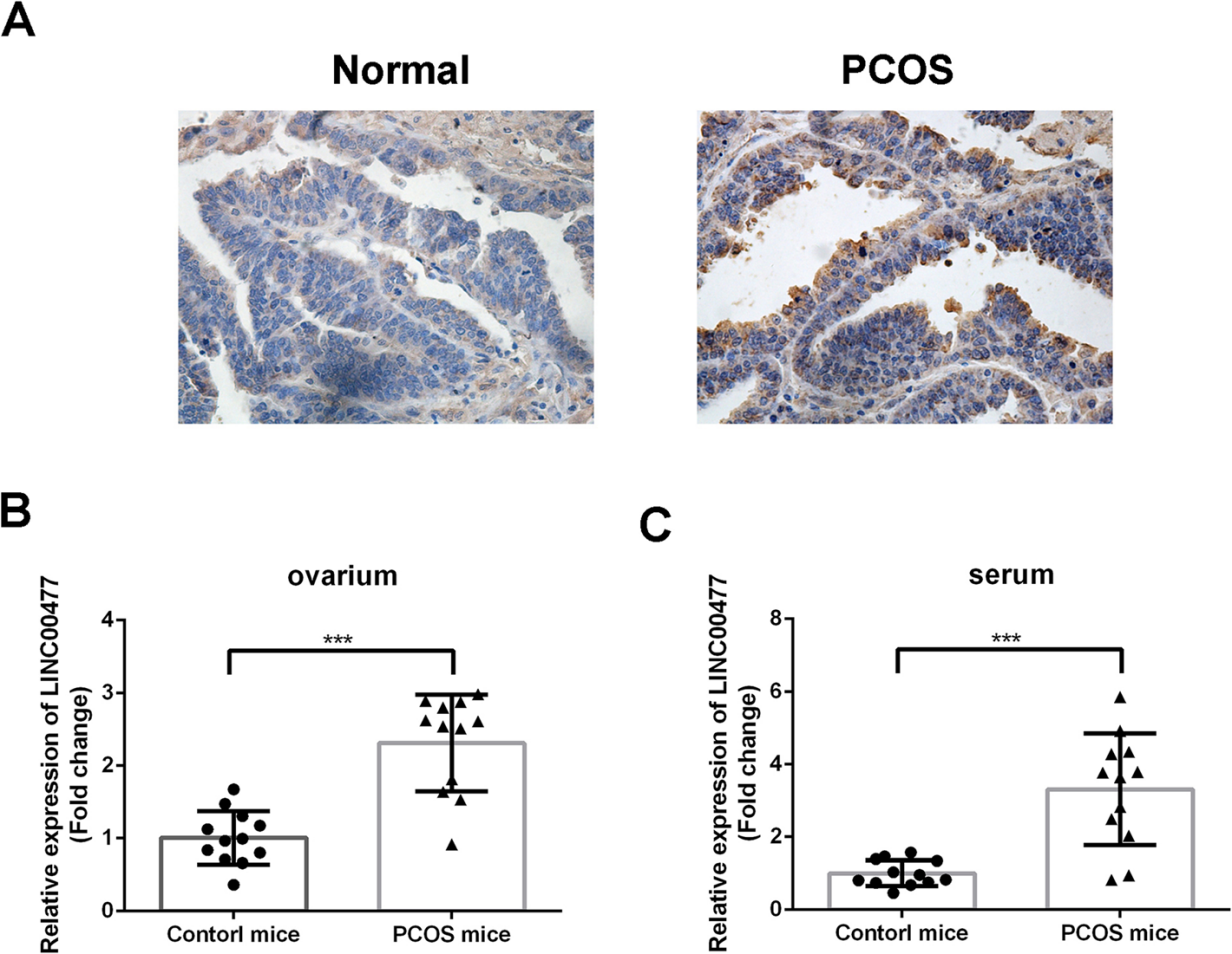
Increased expression of LINC00477 in PCOS in vivo. **a** The ISH staining of LINC00477 in ovarium for PCOS and control mice. LINC00477 was over-expressed in both ovarium (**b**) and serum (**c**) of the PCOS mice compared with the control mice. ***p < 00.01 v.s. control mice

Next, the roles of LINC00477 in PCOS were also explored by mouse PCOS models in vivo. ISH staining of the ovarium of PCOS and control mice is shown in Fig. 2a. LINC00477 was overexpressed in the ovarium of PCOS mice. Moreover, RT-qPCR analysis was performed to compare LINC00477 levels in the ovarium and serum of mice. LINC00477 was significantly increased in both the ovarium (Fig. 2b, *p* < 0.001) and serum (Fig. 2c, *p* < 0.001) of PCOS mice compared with the control mice. The level of LINC00477 is about 2.3 times higher in ovarium and 3.3 times higher in serum of PCOS mice than in the controls.

### LINC00477 affects granulosa cell proliferation and apoptosis in vitro

**Fig. 3:**
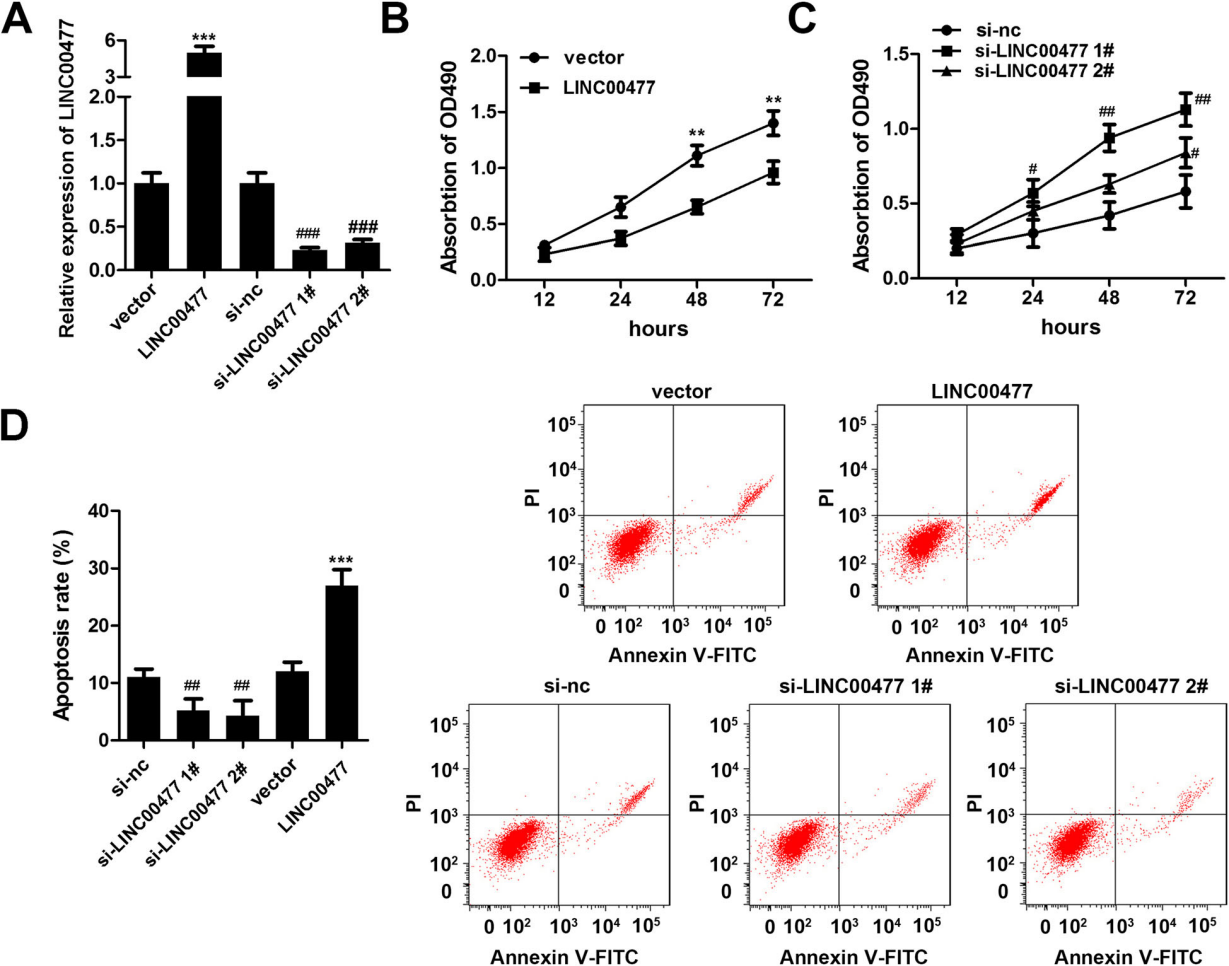
Effect of LINC00477 over-expression plasmid and siRNA on the proliferation and apoptosis of granulosa cells in vitro. **a** Transfection efficiency. **b**-**c** Proliferation of granulosa cells by MTT assay. **d** Apoptosis of granulosa cells by flow cytometry assay. **p < 0.01 v.s. vector, ***p < 0.001 v.s. vector, #*p* < 0.05 v.s. si-nc, ##*p* < 0.01 v.s. si-nc, ###*p* < 0.001 v.s. si-nc

To investigate the roles of LINC00477 in regulating granulosa cell proliferation and apoptosis, cells were treated with LINC00477 overexpression plasmid or siRNA, and the transfection efficiency is shown in Fig. 3a. Compared with the cells transfected with empty vector, the levels of LINC0047 increased more than 5 times in LINC00477 overexpression group; on the other hand, si-LINC00477 1# (Fold change 0.26 v.s. 1, si-LINC00477 1# v.s. si-NC) and 2#(Fold change 0.34 v.s. 1, si-LINC00477 1# v.s. si-NC) markedly decreased the level of LINC00477 in comparison with the si-NC group. Moreover, cell proliferation and apoptosis were determined. We found that the LINC00477 overexpression plasmid dramatically inhibited granulosa cell proliferation at 48 h (Fig. 3b, *p* < 0.01) and 72 h (OD 490 0.80 v.s. 1.41, LINC00477 v.s. vector, Fig. 3b, *p* < 0.01) and promoted apoptosis (Apoptosis rate 27.13 v.s. 10.07, LINC00477 v.s. vector, Fig. 3d, *p* < 0.01) in vitro, whereas knockdown of LINC00477 promoted granulosa cell proliferation at 24 h (OD 490 0.58 v.s. 0.32, si-LINC00477 1# v.s. si-NC, Fig. 3c, *p* < 0.05), 48 h (OD 490 0.91 v.s. 0.43, si-LINC00477 1# v.s. si-NC, Fig. 3c, *p* < 0.01) and 72 h (OD 490 1.04 v.s. 0.50, si-LINC00477 1# v.s. si-NC; 0.84 v.s. 0.50 si-LINC00477 2# v.s. si-NC; Fig. 3c, *p* < 0.01) and inhibited apoptosis (Apoptosis rate 4.18 v.s. 10.03, si-LINC00477 1# v.s. si-NC; 3.76 v.s. 10.03, si-LINC00477 2# v.s. si-NC, Fig. 3d, *p* < 0.01) in vitro. Because si-LINC00477 1# has shown better effects, therefore, si-LINC00477 1# was used in the following experiments.

### miR-128 is a target of LINC00477

**Fig. 4:**
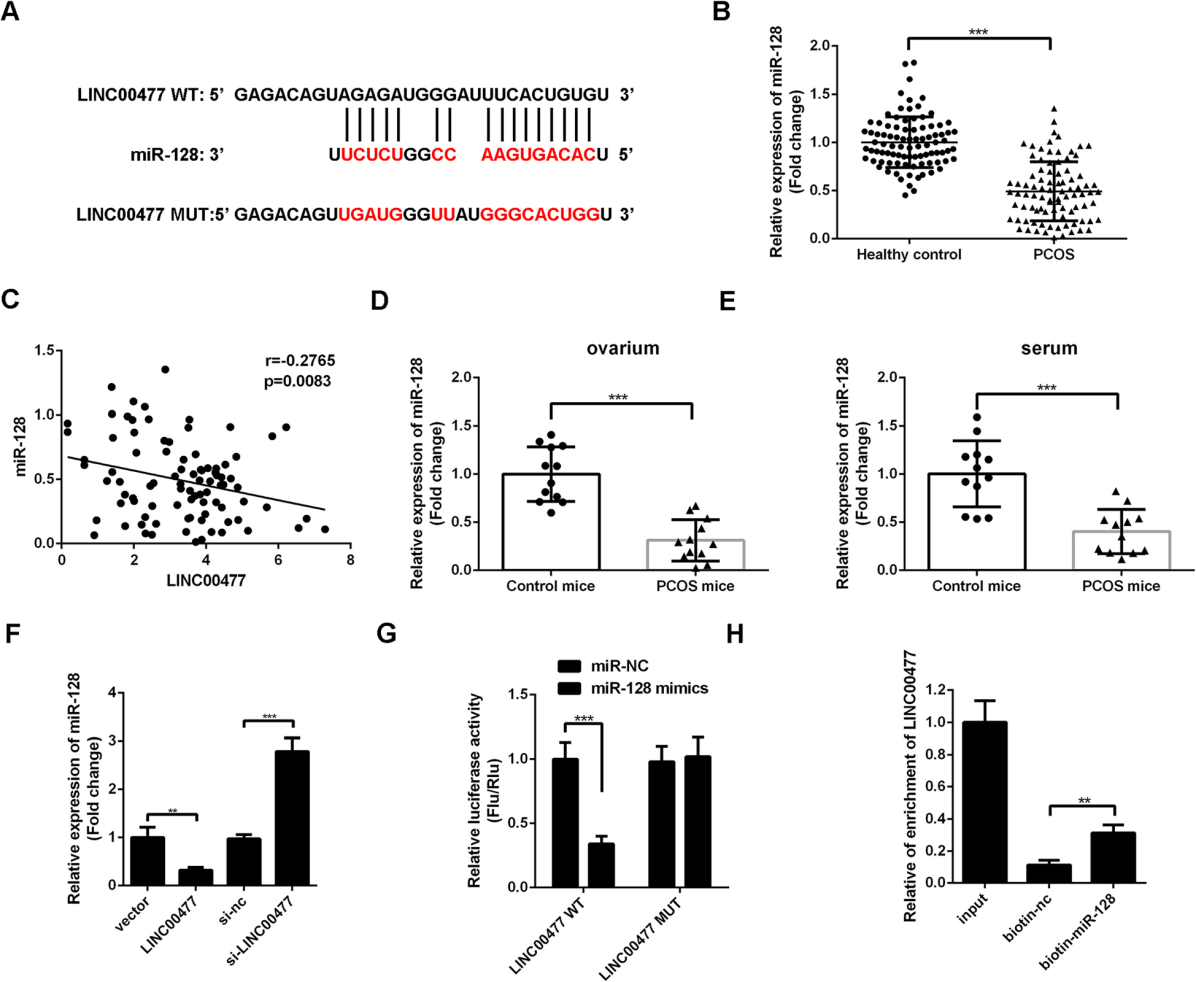
miR-128 is a target of LINC00477. **a** Results of bioinformatic analysis showing the 3′-UTR binding regions of LINC00477 and miR-128. **b** Comparison of the levels of miR-128 in serum of the PCOS patients (*n* = 90) and healthy controls (*n* = 90). **c**Correlation between the levels of miR-128 and LINC00477 in serum of the PCOS patients. **d** Comparison of the levels of miR-128 in ovarium of the control (*n* = 12) and PCOS (*n* = 12) mice. **e** Comparison of the levels of miR-128 in serum of the control (*n* = 12) and PCOS (*n* = 12) mice. **f** Effect of LINC00477 and si-LINC00477 on the expression of miR-128 in granulosa cells. **g** Results of dual-luciferase analysis. **h** Results of RNA pull-down analysis. **p < 0.01, ***p < 0.001. Each cell experiment (**f**-**h**) was repeated for at least 3 times

Next, the underlying mechanism of LINC00477 in PCOS was explored. First, miR-128 was identified as a target of LINC00477 by bioinformatic analysis (Fig. 4a). Next, the expression of miR-128 in the serum of PCOS patients was examined by RT-qPCR. miR-128 levels were markedly decreased in the serum of PCOS patients compared with the control (Fold change 0.49 v.s. 1, PCOS v.s. healthy control, Fig. 4b, *p* < 0.01). Furthermore, correlation analysis results showed that serum levels of LINC00477 and miR-128 were negatively correlated (Fig. 4c, *r* = − 0.2765, *p* = 0.0083). Additionally, miR-128 levels in the ovarium and serum of the PCOS mice were examined. miR-128 was decreased in the ovarium (Fold change 0.31 v.s. 1, PCOS mice v.s. control mice, Fig. 4d, *p* < 0.01) and serum (Fold change 0.4 v.s. 1, PCOS mice v.s. control mice, Fig. 4e, *p* < 0.01) of PCOS mice.

Next, the effects of the LINC00477 overexpression plasmid as well as LINC00477 siRNA on miR-128 expression levels were examined. As shown in Fig. 4f, LINC00477 overexpression markedly decreased miR-128 levels (Fold change 0.32 v.s. 1, LINC00477 v.s. vector, *p* < 0.01), and LINC00477 siRNA significantly increased miR-128 expression in granulosa cells (Fold change 2.79 v.s. 1, si- LINC00477 v.s. si-NC, *p* < 0.001). Finally, a dual-luciferase reporter assay and RNA pull-down assay were performed to evaluate the direct targeting relationship between LINC00477 and miR-128. As shown in Fig. 4g, miR-128 mimics markedly decreased the luciferase activity in LINC00477 wt transfected cells (Relative Flu/Rlu 0.34 v.s. 1, miR-128 mimics v.s. miR-128 NC, *p* < 0.001) but had no significant effects on the luciferase activity of LINC00477 mut transfected cells (Relative Flu/Rlu 1.02 v.s. 0.98, miR-128 mimics v.s. miR-128 NC, *p* > 0.05). On the other hand, RNA pull-down assay results showed that compared with the biotin-NC group, biotin-miR-128 markedly increased LINC00477 expression (Fold change 0.31 v.s. 0.11, biotin-nc v.s. biotin-miR-128, Fig. 4h, *p* < 0.01). These results suggested that LINC00477 can directly target miR-128.

### LINC00477 regulates granulosa cell proliferation and apoptosis by targeting miR-128

**Fig. 5:**
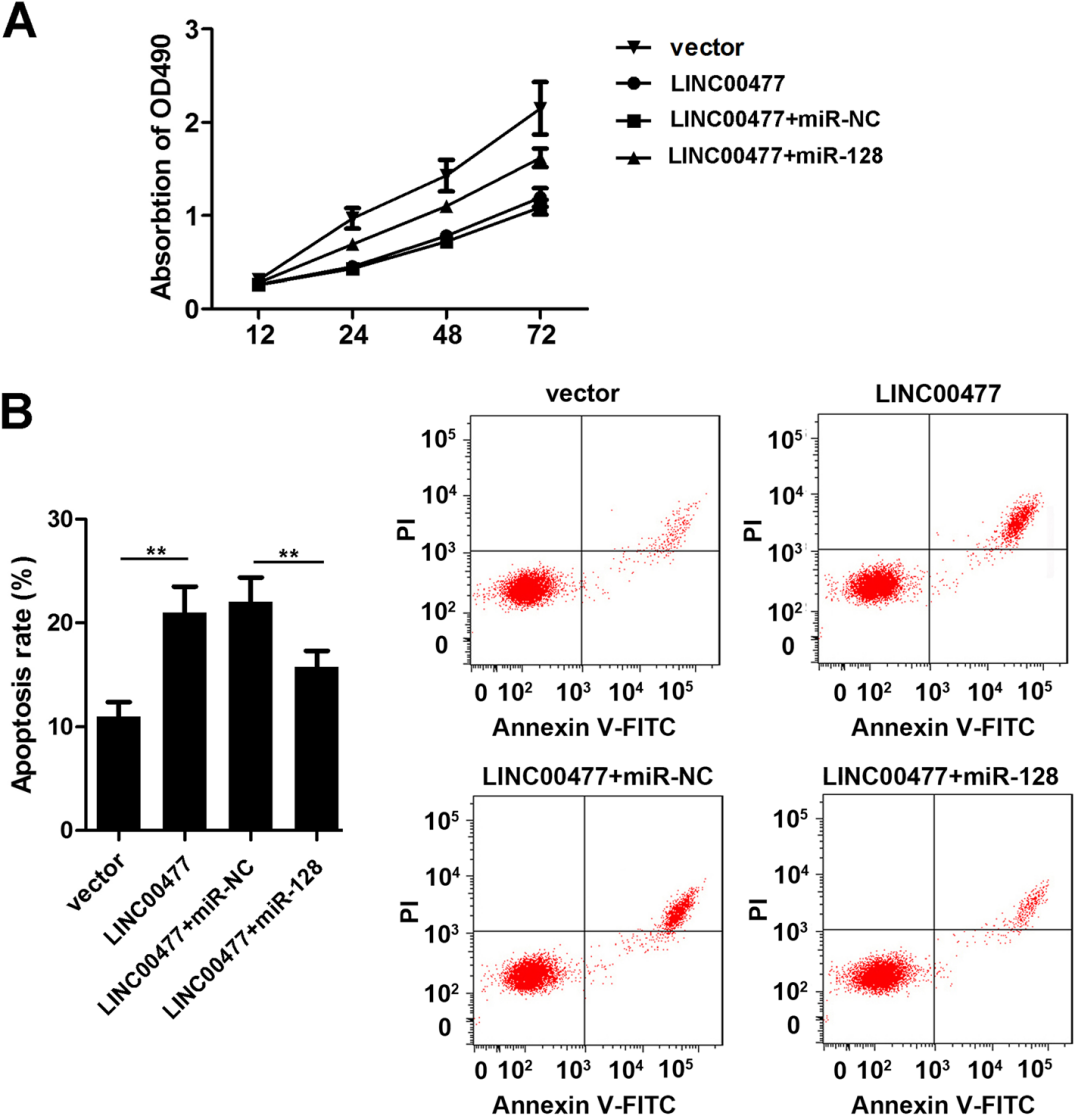
LINC00477 inhibits the proliferation and promote the apoptosis of PCOS cells via targeting miR-128. **a** Proliferation of granulosa cells by MTT assay. **b** Apoptosis of granulosa cells by flow cytometry assay. *p < 0.05, **p < 0.01. Each experiment was repeated for at least 3 times. *P < 0.05, ***P < 0.001

Finally, we cotransfected the LINC00477 overexpression plasmid and miR-128 mimics into granulosa cells, and cell proliferation and apoptosis were determined. As shown in Fig. 5a, compared with the LINC00477 overexpression group, miR-128 mimics partially abrogated the effects of LINC00477 by promoting the proliferation at 48(OD 490 0.56 v.s. 1.01, LINC00477 + miR-NC v.s. LINC00477 + miR-128, Fig. 5a, *p* < 0.01) and 72 h (OD 490 0.89 v.s. 1.38, LINC00477 + miR-NC v.s. LINC00477 + miR-128, Fig. 5a, *p* < 0.01) and inhibiting the apoptosis (Apoptosis rate 22.56 v.s. 16.14, LINC00477 + miR-NC v.s. LINC00477 + miR-128, Fig. 5b and c, *p* < 0.01) of granulosa cells in vitro.

## Discussion

In the present study, the roles of LINC00477 in PCOS and the underlying mechanism were explored. We found that LINC00477 may promote PCOS by inhibiting the proliferation and increasing apoptosis of granulosa cells by modulating miR-128 expression.

The roles of lncRNAs in PCOS have been discussed in many previous studies. For example, Zhu et al. suggested that knockdown of lncRNA ZFAS1 increases granulosa cell proliferation and inhibits apoptosis in PCOS [18]. Liu et al. found that the lncRNA PVT1/microRNA-17-5p/PTEN axis regulates E2 and P4 secretion and granulosa cell proliferation and apoptosis in PCOS [14]. Chen et al. found that lncRNA HCP5 promotes cell proliferation and inhibits apoptosis via the miR-27a-3p/IGF-1 axis in the human granulosa-like tumor cell line KGN [19]. LINC00477 is a recently identified lncRNA, and Zhao et al. reported the role of LINC00477 in gastric cancer [16]. In a recent sequencing analysis, LINC00477 was identified as one of the most significantly upregulated lncRNAs in PCOS patients [17]; however, this result has not yet been verified using a large number of clinical samples. In this study, we observed increased LINC00477 expression in the serum of PCOS patients, which was consistent with the sequencing results. Interestingly, ROC analysis results showed that LINC00477 serum levels can sensitively distinguish PCOS patients from healthy controls. Moreover, we also confirmed the overexpression of LINC00477 in PCOS animal models. Taken together, these data suggested that LINC00477 was upregulated in PCOS. However, the underlying mechanism still requires further investigation.

Aberrant granulosa cell apoptosis is an important reason for the development of PCOS [20–22]. In the present study, we found that LINC00477 overexpression inhibited granulosa cell proliferation and apoptosis. LINC00477 knockdown showed the opposite effects. These results suggested that LINC00477 may serve as a potential novel therapeutic for targeted therapy for PCOS.

In previous studies, the interaction between lncRNAs and miRNAs was identified as the most common underlying mechanism by which lncRNAs exert their biological functions by “sponging” and silencing target miRNAs [23–25]. With the development of bioinformatics, the target miRNAs of lncRNAs can be predicted using various methods. We used the online tool starBase 3.0, and miR-128 was identified as a target miRNA of LINC00477. We found that miR-128 was a target of LINC00477. Therefore, we hypothesized that LINC00477 may participate in PCOS by downregulating miR-128 expression. To verify this hypothesis, we performed a series of experiments. First, miR-128 expression levels in patient sera was examined, and we confirmed the negative correlation between miR-128 and LINC00477 levels in PCOS patient sera; moreover, miR-128 expression was downregulated in the ovarium and the serum of PCOS mice, which was consistent with its expression pattern in the PCOS patient serum. Moreover, the direct targeting relationship between LINC00477 and miR-128 was also confirmed by dual luciferase reporter assays as well as RNA pull-down assays. Interestingly, the results of cell experiments suggested that miR-128 mimics partially abrogated the effects of the LINC00477 overexpression plasmid on granulosa cell proliferation and apoptosis. Taken together, these results demonstrated that upregulation of LINC00477 may facilitate the development of PCOS by decreasing miR-128 expression.

In summary, we reported for the first time that LINC00477 may regulate the proliferation and apoptosis of granulosa cells by modulating miR-128 expression. Our data suggested that targeting the LINC00477/miR-128 axis may represent a potential method for the treatment of PCOS.

## Data Availability

All data generated or analyzed during this study are included in this published article or are available from the corresponding author on reasonable request.
